# Datasets for high hydrogen content syngas fuel variability effect on combustion physicochemical properties

**DOI:** 10.1016/j.dib.2020.105116

**Published:** 2020-01-11

**Authors:** Kai Zhang, Xi Jiang

**Affiliations:** a Linné Flow Center, Department of Mechanics, Royal Institute of Technology (KTH), Osquars Backe 18, SE-10044, Stockholm, Sweden; b School of Engineering & Materials Science, Queen Mary University of London, Mile End Road, London, E1 4NS, UK

**Keywords:** UQ, Combustion, Syngas, Sensitivity analysis, Fuel variability

## Abstract

The dataset presented in this article is related to the uncertainty quantification of fuel variability effect on high hydrogen content syngas combustion physicochemical properties. The 1D flame data included in this dataset are collected using PREMIX module available in Chemkin-Pro. Inputs to and outputs collected from the PREMIX module are generated and post-processed using UQTk-3.0.4, an open-access uncertainty quantification (UQ) toolkit developed at Sandia National Laboratories. The 1D flame data here refers to the calculation of flame speed, flame temperature, NO emission, *etc*. using three detailed chemical mechanisms: the GRI-Mech 3.0, the San Diego, and the NUI Galway Mechanism. The main analysis performed using UQTk-3.0.4 focuses on obtaining main and joint sensitivity effects (Sobol Indices) of uniformly distributed fuel uncertainty on 1D premixed physicochemical property. Other parameters such as the resulted probability density function or fluctuation of these properties are also explored. This new and original dataset is suitable for further analyzing fuel variability effect on other significant flame controlling parameters such as Karlovitz number, flame thickness, *etc.* in the discipline of turbulent combustion simulation.

**Table:** Specifications Table

Subject	Energy
Specific subject area	Energy Engineering and Power Technology; Renewable Energy, Sustainability and the Environment
Type of data	Table and Figures
How data were acquired	Numerical calculation of 1D premixed flame in PREMIX module available in Chemkin-Pro [1], pre- and post-process flame data in UQTk-3.0.4 [2], an open-access uncertainty quantification toolkit developed at Sandia National Laboratories. Three detailed chemical mechanisms: the GRI-Mech 3.0 [3], the San Diego [4], and the NUI Galway Mechanism [5] are used for calculating 1D flame physicochemical properties at various conditions. Other Python features are added to UQTk scripts to generate figures reported in this article.
Data format	Raw and analysis
Parameters for data collection	Four cases are analyzed namely HXXCOXX, *e.g.* H60CO30 represents a syngas mixture with 60%H_2_, 30%CO in addition to the other two species with their mole fraction always set to constant: 5.5%CO_2_ and 4.5%CH_4_. Flame physicochemical properties at 6 equivalence ratios are calculated using PREMIX: 0.45, 0.5, 0.55, 0.6, 0.8, 1.0. Unburnt gas temperature and pressure are set at 400K, 1atm.
Description of data collection	Numerical calculation of 1D flame properties invoking pre- and post- statistical analysis.
Data source location	Institution: Queen Mary University of London City/Town/Region: London Country: United Kingdom
Data accessibility	Repository name: Mendeley data Direct URL to data: https://doi.org/10.17632/67854cyf78.1
Related research article	Zhang, K. and Jiang, X., 2019. Uncertainty quantification of fuel variability effects on high hydrogen content syngas combustion. *Fuel*, *257*, p.116,111. https://doi.org/10.1016/j.fuel.2019.116111

**Table:** 

**Value of the Data** • Uncertainty of syngas fuel variability often induces unstable operation issues of gas turbines. A quantified impact could help reduce such unstable problems by minimizing the uncertainty of dominating species from upstream gasification process. • Power plants, gas turbine engineers can benefit from these data. • Probability density functions (PDFs) for flame quantities are constructed via pushing 1,000,000 sample points into a constructed surrogate model. These data points can be further processed to understand the risky conditions of operating gas turbines, *e.g.* to calculate Karlovitz number which has an indication of flame stability in Borghi diagram [6]. • Many controlling parameters have been used in the presented datasets such as equivalence ratio, hydrogen content, *etc.* to construct surrogate models that can replace ODEs in 1D premixed code. The massive quantified impact of uncertainty on flame physicochemical properties such as CO_2_, CO, NO, *etc.* is of great importance in practical gas turbine operation.

## 1 Data description

Here we describe part of the results generated with our datasets to help clarify the data structure. Some of the pre- and post-processing techniques are also described for ease to understand how data are distributed in each data consisting directory. The example figures produced from our datasets deal with that obtained using the GRI-Mech 3.0 mechanism. The data structure is the same for those obtained with other mechanism.

Four high hydrogen content syngas fuel mixtures are assumed namely H60CO30, H50CO40, H40CO50, and H30CO60, giving an example that H60CO30 represents a syngas-hydrogen mixture with 60%H_2_, 30%CO in addition to the other two fixed compositions of 5.5%CO_2_ and 4.5%CH_4_. Each composition except CH_4_ is assumed to contain a small variation of 1.5%, *e.g.*, CO_2_ composition can vary from 4% to 7% in H60CO30, H_2_ can vary from 58.5% to 61.5% with their actual value sampled from a uniformly distributed PDF. The effect of these changes on flame physicochemical properties is of great importance, and hence uncertainty quantification method is employed for the investigation. Details of the UQ are described in the following section. The sampled data from assumed uniform PDFs are pre-processed based on a deterministic sampling method that instead of random sampling over the entire uncertain space, only typical points are sampled.

The sampled (or pre-processed) data are stored with explicit file name given as: ‘Xch4_sample.txt’, ‘Xco2_sample.txt’, ‘Xco_sample.txt’, and ‘Xh2_sample.txt’. These files can be found in any of the subfolders of the directory ‘Data/Datasets/1atm_GRI-Mech 3.0_UQ/HXXCOXX/Eq = XXX’ where XX represents the H_2_ and CO mole fraction as described above, and XXX represents the equivalence ratios. For a typical HXXCOXX, though the four sampled data files can always be found in any of its sub-folders of ‘Eq = XXX’, the file with the same name always share the same content.

These sampled points are then passed through 1D premixed code in Chemkin to output flame physicochemical property data stored in the directory ‘Data/Datasets/1atm_GRI-Mech 3.0_PREMIX/HXXCOXX/’. The data are named in the form ‘Y_sample_YY.txt’, where Y refers to quantity such as adiabatic flame temperature (T), flame speed (S), NO, CO_2_, and NO_2_ emissions (NO, CO_2_, NO_2_), YY represents the equivalence ratio at which these flame quantities are calculated, *e.g.* YY = 055, 06 and 10 represent equivalence ratio of 0.55,0.6 and 1.0 respectively. These flame quantities are then passed back to the directory which stores sampled (or pre-processed) data according to the given subfolders name defined and are used for post-processing in UQTk 3.0.4. For instance, the NO_sample_055.txt is passed to the subfolder ‘NO’ in the directory ‘Data/Datasets/1atm_GRI-Mech 3.0_UQ/HXXCOXX/Eq = 0.55’. All post-processed data, though stored in the same directory, are re-collected into other top-level directories for plotting useful figures with examples given as follows.

**Fig. 1:**
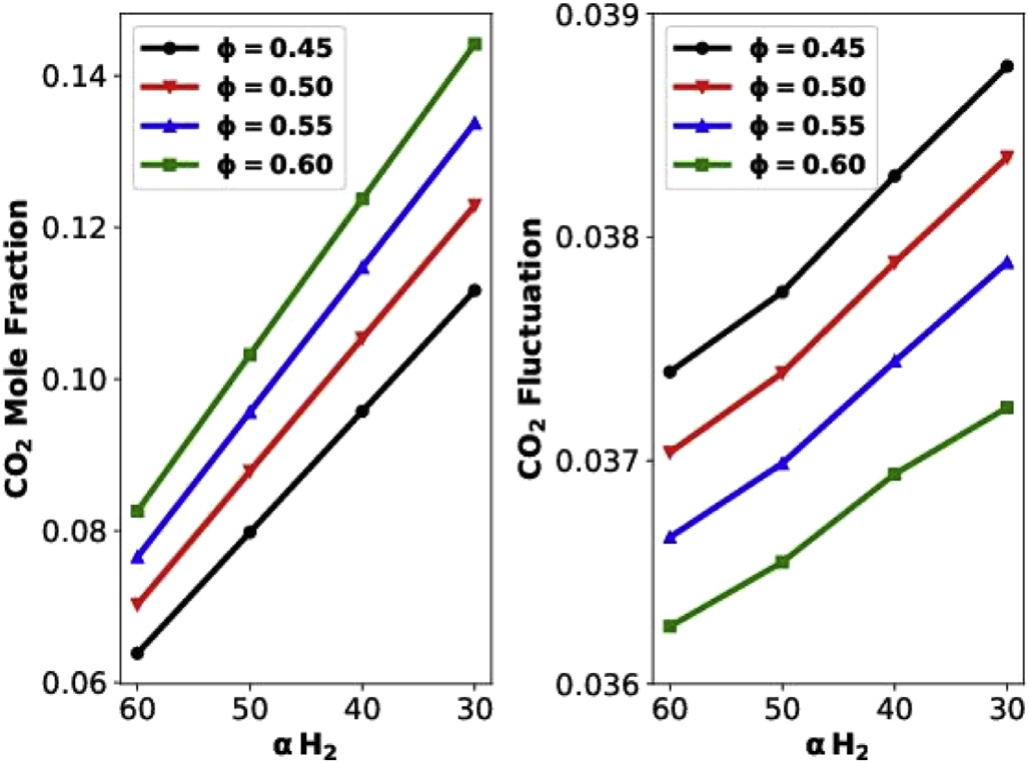
Data_Figures_GRI-Mech _CO2_HCOratio.

In the following, the ‘Fig. 1. Data_Figures_GRI-Mech _CO2_HCOratio’ refers to the figures created using post-processed data in the following directory: ‘Data/Figures/GRI-Mech/CO2_HCOratio/’. The folder name ‘CO2_HCOratio’ means the data contained in the folder is for CO_2_ emission (y-axis) with respect to H_2_:CO ratio (x-axis). The file name in that directory is formatted as ‘XX_CO2_X.txt’ where XX is the mean or variance of CO_2_, X can be 0, 1, 2, or 3 representing H60CO30, H50CO40, H40CO50, and H30CO60 in sequential order. Each file contains 6 rows of data representing the flame quantity at 6 equivalence ratios: 0.45, 0.5, 0.55, 0.6, 0.8 to 1.0.

**Fig. 2:**
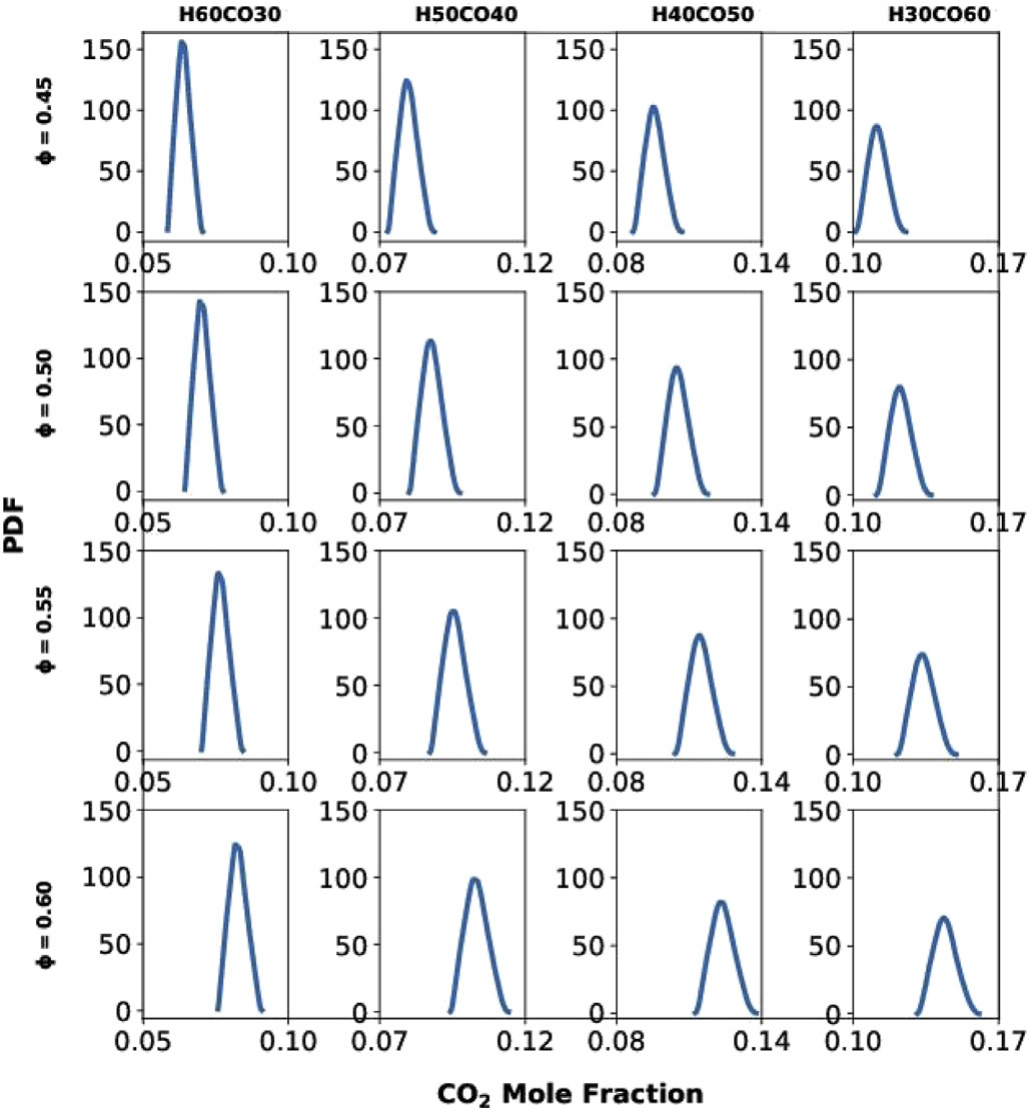
Data_Figures_GRI-Mech_CO2_PDF.

The ‘Fig. 2. Data_Figures_GRI-Mech_CO2_PDF’ refers to the figure created using data in the following directory: ‘Data/Figures/GRI-Mech/CO2_PDF/’. The folder name ‘CO2_PDF’ means the data contained in the folder is for CO_2_ emission (x-axis) versus PDF (y-axis). The file name in that directory is formatted as PDF_X_axis_XX_XXX.txt where X represents x or y (data) of the figure, XX represents equivalence ratio, XXX indicates typical syngas-hydrogen mixture.

**Fig. 3:**
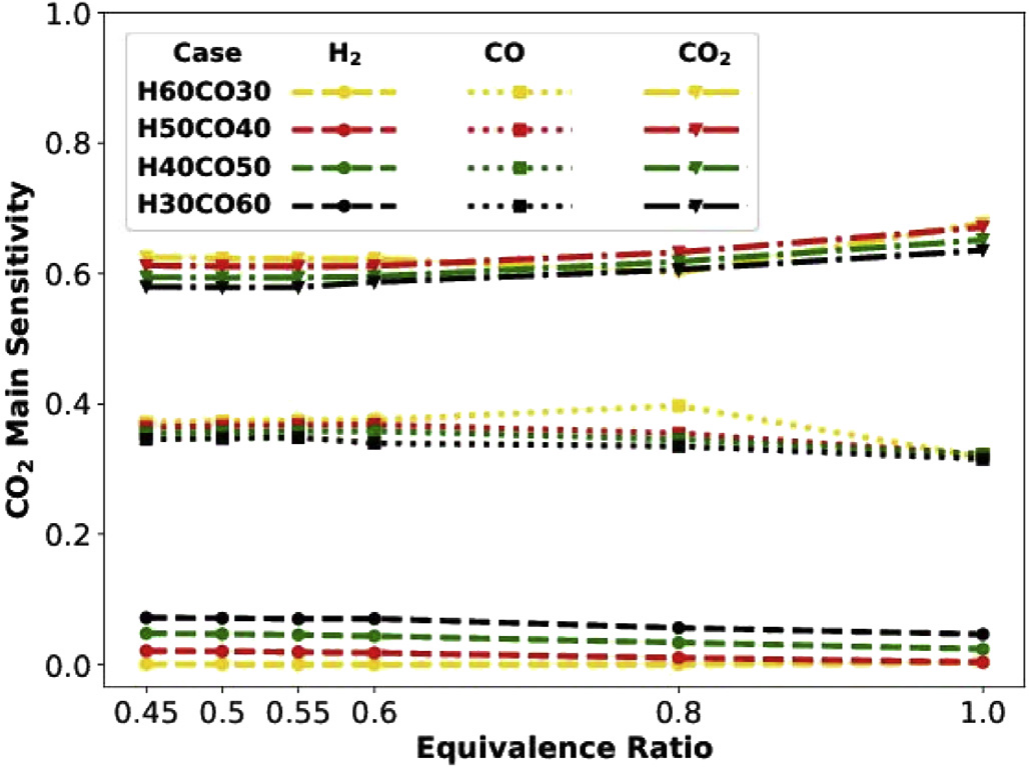
Data_Figures_GRI-Mech _CO2main.

**Fig. 4:**
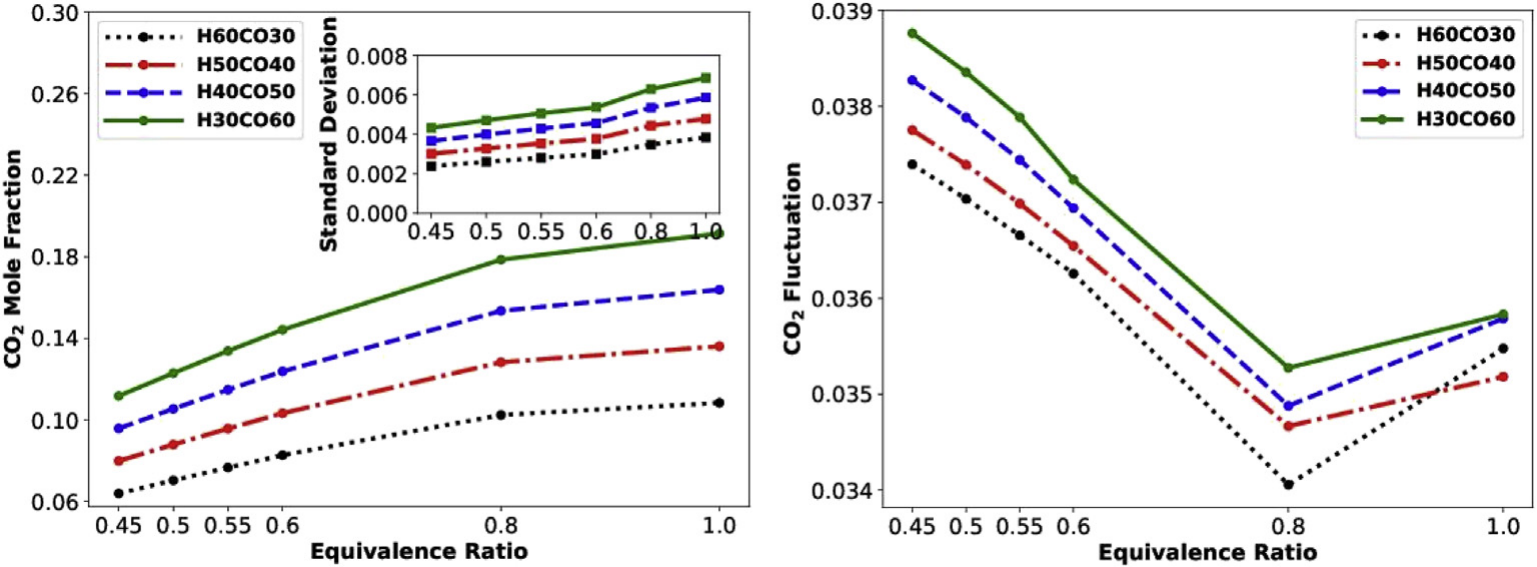
Data_Figures_GRI-Mech _CO2meanstd.

Hereafter we only describe figures without referring to the relevant directories unless it is exceptionally necessary. Fig. 3 shows the main sensitivity of CO_2_ emission with respect to different mixture compositions. Three rows of data are available in each file, representing the main sensitivity of CO_2_ emission with respect to H_2_, CO, and CO_2_ variability in the syngas-hydrogen mixture. Fig. 4 shows mean, standard deviation, and fluctuation of CO_2_ emission. The fluctuation is defined as mean divided by standard deviation (square root of variance). Each row in the data file associates with a typical equivalence ratio.

**Fig. 5:**
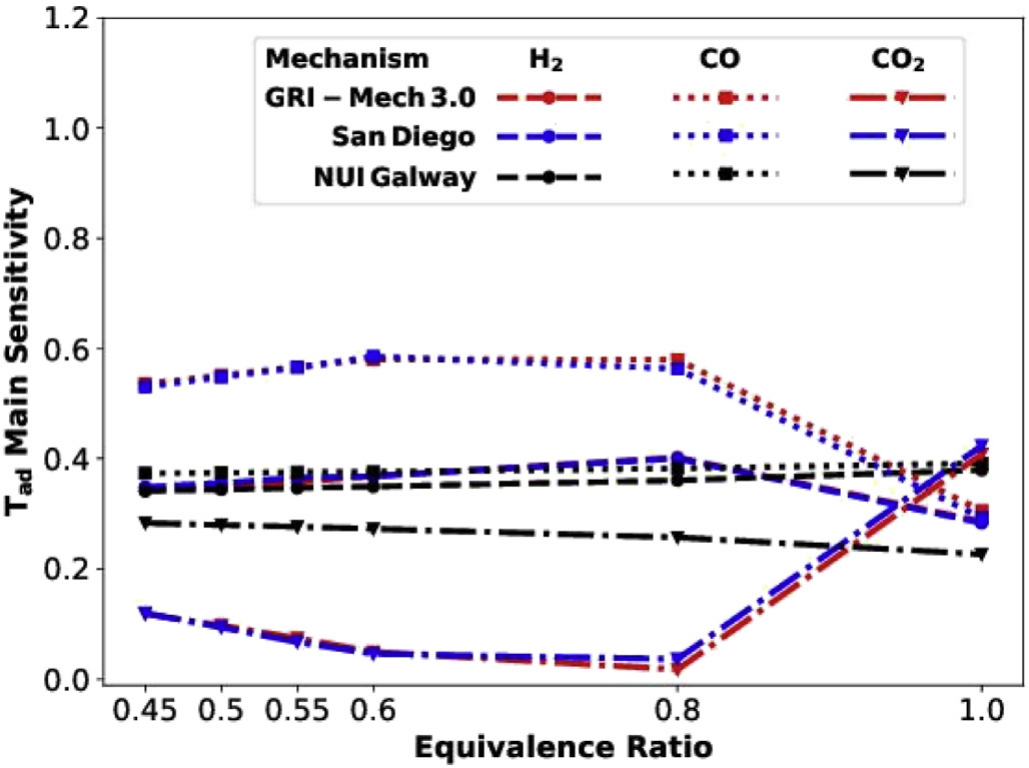
Data_Figures_Cross_comparison _Tmain.

The above-mentioned data structure also applies to those generated with NUI_Galway and San_Diego mechanism. Next, there is a special folder called ‘Cross_comparison’ in the directory ‘Data/Figures/’. Folder names are formatted in a similar way as that given above, the only exception is that the file names given in the folder are added with either ‘_N’, ‘_G’ or ‘_S’, each representing data collected from ‘NUI-Galway’, ‘GRI-Mech’, and ‘San_Diego’ folder in the directory ‘Data/Figures/’. Fig. 5 shows the main sensitivity comparison of adiabatic flame temperature data obtained using different chemical mechanisms.

## 2 Experimental design, materials, and methods

A PCE based surrogate model established based on a deterministic sampling (DM) method, is chosen to quantify the effect of high hydrogen content syngas fuel variability effect on its combustion thermophysical properties. The DM is recognized to converge faster than Monte-Carlo sampling (MCS) or Latin-Hypercube-Sampling (LHS) method [7]. The PCE is built for the convenience of performing variance-based sensitivity analysis (Sobol Indices [8]), that is, to answer the question of how the output uncertainty is affected by the input uncertainty on a variance base. Details of the PCE-DM are introduced below.

A general PCE representing a one-dimensional (1D) random variable (RV) u can be written as,

(1) $$u=\sum_{k=0}^{P}C_{k}He_{k}\left(\xi \right)$$

where $C_{k}$ is the PC coefficient, $He_{k}$ is 1D Hermite polynomial of order *P*, and ξ is Gaussian RV in the range [−∞,∞]. The equation expresses a single variable as a set of Hermite functions ${He_{k}\left(\xi \right)=\left(-1\right)}^{k}e^{\xi^{2}/2}\left(d^{k}/d\xi^{k}\right)e^{-\xi^{2}/2}$ multiplied with deterministic PC coefficients $C_{k}$ (refer to ‘c_k.txt’ in the directory where post-processing is performed). The Hermite polynomials are orthogonal with respect to the weight function $w\left(\xi \right)=\left(1/\sqrt{2\pi }\right)e^{-\xi^{2}/2}$, giving,

(2) $$\langle He_{j}He_{k}\rangle =\int_{-\infty }^{\infty }He_{j}\left(\xi \right)He_{k}\left(\xi \right)w\left(\xi \right)d\xi =\delta_{jk}\langle He_{k}^{2}\rangle$$

where $\delta_{jk}$ is the Kronecker delta, and <> represents statistical expectation. For all j≠k, the expectation of the product of any two polynomials equals to zero. For j=k and $u\approx \overset{P}{\underset{k=0}{\sum }}C_{k}He_{k}$, the following expression for PC coefficient can be obtained from $\langle He_{k}u\rangle =\overset{P}{\underset{k=0}{\sum }}C_{k}\langle He_{k}He_{k}\rangle =C_{k}<He_{k}^{2}>$ and $\langle uHe_{k}\rangle =\overset{\infty }{\underset{-\infty }{\int }}uHe_{k}\left(\xi \right)w\left(\xi \right)d\xi$ (re-arrange equation (2)), giving,

(3) $$C_{k}=\frac{<uHe_{k}>}{<He_{k}^{2}>}=\frac{1}{<He_{k}^{2}>}\int_{-\infty }^{\infty }uHe_{k}\left(\xi \right)w\left(\xi \right)d\xi$$

where the denominator can be pre-calculated, and the integral is evaluated using numerical quadrature rules (referred to as DM method), written as,

(4) $$\overset{\infty }{\underset{-\infty }{\int }}uHe_{k}\left(\xi \right)w\left(\xi \right)d\xi =\sum_{i=1}^{N_{q}}q^{i}u\left(\xi^{i}\right)He_{k}\left(\xi^{i}\right)$$

where $q^{i}$ is the corresponding weights, and $N_{q}$ is the number of quadrature points needed for accurate integration. For multi-dimensional (*D*) Hermite polynomial of order *P*, number of quadrature points ${N_{q}=\left(p+1\right)}^{D}$.

Assuming there exist three input uncertainties giving *D* = 3 which are associated with Gaussian PDF, and assuming a 5th order Hermite polynomial is required to replace a typical numerical model, equation (1) can be written as,

(5) $$Q\left(\lambda_{1},\lambda_{2},\lambda_{3}\right)=\sum_{k=0}^{5}C_{k}He_{k}\left(\xi_{1},\xi_{2},\xi_{3}\right)=C_{0}He_{0}\left(\xi_{1},\xi_{2},\xi_{3}\right)+C_{1}He_{1}\left(\xi_{1},\xi_{2},\xi_{3}\right)+\ldots +C_{5}He_{5}\left(\xi_{1},\xi_{2},\xi_{3}\right)$$

where the $He_{k}\left(\xi_{1},\xi_{2},\xi_{3}\right)$ is a multi-dimensional Hermite polynomial, the Q represents the interested quantity such as thermophysical and transport properties of the high hydrogen content syngas fuel at certain conditions. The $\lambda_{1},\lambda_{2},\lambda_{3}$ (refers to sampled or pre-processed data) represent the three uncertain model parameters which can also be expanded individually using PCE as,

(6) $$\text{λ}\left(\xi \right)=\sum_{k=0}^{P}C_{k}He_{k}\left(\xi \right)$$

Because it is assumed that the three input parameters have uncertainty that is normally distributed, first-order Hermite polynomial gives the following expression:

(7) $$\text{λ}\left(\xi \right)=C_{0}He_{0}\left(\xi \right)+C_{1}He_{1}\left(\xi \right)=C_{0}\times 1+C_{1}\times \text{ξ}=\text{μ}+\text{σ}\xi$$

where $C_{0}=\text{μ}$ (expectation) and $C_{1}=\text{σ}$ (standard deviation). The germ samples ξ are randomly sampled from [−∞,∞] (refer to ‘qdpts.txt’ in the directory where post-processing is performed). Substitute equation (7) into (5), the quantity *Q* is decomposed to the form of PCE.

Using PCE based surrogate model provides ease of achieving sensitivity information (effect of input uncertainty on that of output) from decomposed terms of polynomial chaos expansion. For example, equation (5) can be further expanded as,

(8) $$Q\left({\text{μ}}_{1}+{\text{σ}}_{1}\xi_{1},{\text{μ}}_{2}+{\text{σ}}_{2}\xi_{2},{\text{μ}}_{3}+{\text{σ}}_{3}\xi_{3}\right)=C_{0}+C_{1}He_{1}\left(\xi_{1}\right)+C_{2}He_{1}\left(\xi_{2}\right)+C_{3}He_{1}\left(\xi_{3}\right)+C_{4}He_{2}\left(\xi_{1}\right)+C_{5}He_{1}\left(\xi_{1}\right)He_{1}\left(\xi_{2}\right)+C_{6}He_{1}\left(\xi_{1}\right)He_{1}\left(\xi_{3}\right)+C_{7}He_{2}\left(\xi_{2}\right)+C_{8}He_{1}\left(\xi_{2}\right)He_{1}\left(\xi_{3}\right)+C_{9}He_{2}\left(\xi_{3}\right)+\ldots$$

where each term corresponds to the decomposed effect of a germ ξ on *Q*. Moreover, global sensitivity information can be obtained because the total variance of a targeted quantity equals to the sum of the variance contribution from decomposed terms, *i.e.*, $Var\left(Q\right)=\underset{k>0}{\sum }C_{k}^{2}\langle He_{k}^{2}\rangle$, or,

(9) $$Var\left[Q\left({\text{μ}}_{1}+{\text{σ}}_{1}\xi_{1},{\text{μ}}_{2}+{\text{σ}}_{2}\xi_{2},{\text{μ}}_{3}+{\text{σ}}_{3}\xi_{3}\right)\right]=0+C_{1}^{2}\langle He_{1}^{2}\left(\xi_{1}\right)\rangle +C_{2}^{2}\langle He_{1}^{2}\left(\xi_{2}\right)\rangle +C_{3}^{2}\langle He_{1}^{2}\left(\xi_{3}\right)\rangle +C_{4}^{2}\langle He_{2}^{2}\left(\xi_{1}\right)\rangle +C_{5}^{2}He_{1}^{2}\left(\xi_{1}\right)\langle He_{1}^{2}\left(\xi_{1}\right)\rangle +C_{6}^{2}\langle He_{1}^{2}\left(\xi_{1}\right)\rangle \langle He_{1}^{2}\left(\xi_{3}\right)\rangle +C_{7}^{2}\langle He_{2}^{2}\left(\xi_{2}\right)\rangle +C_{8}^{2}\langle He_{1}^{2}\left(\xi_{2}\right)\rangle \langle He_{1}^{2}\left(\xi_{3}\right)\rangle +C_{9}^{2}\langle He_{2}^{2}\left(\xi_{3}\right)\rangle +\ldots$$

The second and the fifth term of equation (9) represents the variance contribution of $\xi_{1}$ to the variance of the quantity *Q*. In other words, the uncertainty effect of input on total variance of output can be written as,

(10) $$S_{i}=\frac{\sum_{i}C_{i}^{2}\langle \varphi_{i}^{2}\rangle }{\sum_{k>0}C_{k}^{2}\langle \varphi_{k}^{2}\rangle }$$

where *i* refers to one of the three input parameters. In the given datasets, a total of $N_{q}$ quadrature points are employed for PCE surrogate model construction, 1,000,000 points are pushed through the PCE model to ensure a high representation of PCE characters and to construct PDFs described before.

## References

[1] Chemkin | Reaction Design (2019). http://www.reactiondesign.com/products/chemkin/.

[2] Debusschere B.J., Habib N.N., Philippe P.P., Omar M.K., Roger G.G., Olivier P. (2004). Numerical challenges in the use of polynomial chaos representations for stochastic processes. SIAM J. Sci. Comput..

[3] GRI Mech 3.0 (2019). http://combustion.berkeley.edu/gri-mech/version30/text30.html.

[4] Chemical-Kinetic Mechanisms for Combustion Applications, San Diego Mechanism (2019). Mechanical and Aerospace Engineering (Combustion Research). http://combustion.ucsd.edu.

[5] Zhang Y.J., Olivier M., Eric L.P., Gilles B., Henry J.C. (2017). Assessing the predictions of a NOx kinetic mechanism on recent hydrogen and syngas experimental data. Combust. Flame.

[6] Borghi R. (1985). On the structure and morphology of turbulent premixed flames. Recent Advances in the Aerospace Sciences.

[7] Loh W.L. (1996). On Latin hypercube sampling. Ann. Stat..

[8] Sobol I.M. (1990). On sensitivity estimation for nonlinear mathematical models. Matematicheskoe Model..

